# Could Endothelin-1 Be a Promising Neurohormonal Biomarker in Acute Heart Failure?

**DOI:** 10.3390/diagnostics13132277

**Published:** 2023-07-05

**Authors:** Bianca-Ana Dmour, Alexandru Dan Costache, Awad Dmour, Bogdan Huzum, Ștefania Teodora Duca, Adriana Chetran, Radu Ștefan Miftode, Irina Afrăsânie, Cristina Tuchiluș, Corina Maria Cianga, Gina Botnariu, Lăcrămioara Ionela Șerban, Manuela Ciocoiu, Codruța Minerva Bădescu, Irina Iuliana Costache

**Affiliations:** 1 Department of Internal Medicine, “Grigore T. Popa” University of Medicine and Pharmacy, 700115 Iași, Romania; gherasimbianca93@gmail.com (B.-A.D.); dan-alexandru.costache@umfiasi.ro (A.D.C.); stefaniateodoraduca@gmail.com (Ș.T.D.); adriana.ion@umfiasi.ro (A.C.); minerva.badescu@umfiasi.ro (C.M.B.); irina.costache@umfiasi.ro (I.I.C.); 2 Department of Cardiovascular Rehabilitation, Clinical Rehabilitation Hospital, 700661 Iași, Romania; 3 Department of Orthopedics and Traumatology, “Grigore T. Popa” University of Medicine and Pharmacy, 700115 Iași, Romania; awaddmour@gmail.com; 4 Department of Orthopaedics and Traumatology, “St. Spiridon” County Clinical Emergency Hospital, 700111 Iași, Romania; bogdan.huzum93@gmail.com; 5 Department of Physiology, “Grigore T. Popa” University of Medicine and Pharmacy, 700115 Iași, Romania; ionela.serban@umfiasi.ro; 6 Cardiology Clinic, “St. Spiridon” County Clinical Emergency Hospital, 700111 Iași, Romania; 7 Department of Microbiology, “Grigore T. Popa” University of Medicine and Pharmacy, 700115 Iași, Romania; cristina.tuchilus@umfiasi.ro; 8 Microbiology Laboratory, “St. Spiridon” County Clinical Emergency Hospital, 700111 Iași, Romania; 9 Immunology Laboratory, “St. Spiridon” County Clinical Emergency Hospital, 700111 Iași, Romania; ccianga@hotmail.com; 10 Department of Immunology, “Grigore T. Popa” University of Medicine and Pharmacy, 700115 Iași, Romania; 11 Unit of Diabetes, Nutrition and Metabolic Diseases, “Grigore T. Popa” University of Medicine and Pharmacy, 700115 Iași, Romania; ginabotnariu66@gmail.com; 12 Clinical Center of Diabetes, Nutrition and Metabolic Diseases, “St. Spiridon” County Clinical Emergency Hospital, 700111 Iași, Romania; 13 Department of Morpho-Functional Sciences II, “Grigore T. Popa” University of Medicine and Pharmacy, 700115 Iași, Romania; manuela.ciocoiu@umfiasi.ro; 14 Internal Medicine Clinic, “St. Spiridon” County Clinical Emergency Hospital, 700111 Iași, Romania

**Keywords:** endothelin-1, acute heart failure, biomarkers, neurohormonal activation

## Abstract

Acute heart failure (AHF) is a life-threatening condition with high morbidity and mortality. Even though this pathology has been extensively researched, there are still challenges in establishing an accurate and early diagnosis, determining the long- and short-term prognosis and choosing a targeted therapeutic strategy. The use of reliable biomarkers to support clinical judgment has been shown to improve the management of AHF patients. Despite a large pool of interesting candidate biomarkers, endothelin-1 (ET-1) appears to be involved in multiple aspects of AHF pathogenesis that include neurohormonal activation, cardiac remodeling, endothelial dysfunction, inflammation, atherosclerosis and alteration of the renal function. Since its discovery, numerous studies have shown that the level of ET-1 is associated with the severity of symptoms and cardiac dysfunction in this pathology. The purpose of this paper is to review the existing information on ET-1 and answer the question of whether this neurohormone could be a promising biomarker in AHF.

## 1. Introduction

Heart failure (HF) is currently a major public health concern, affecting more than 25 million people worldwide, with a growing prevalence and extensive socio-economic burden [1,2]. Remarkable advances in the past three decades in optimizing the prevention, diagnosis and treatment of HF have contributed to the increase in life expectancy in these patients. However, acute heart failure (AHF) is the most common cause of hospitalization, and the prognosis of patients after discharge continues to be poor [1,3]. AHF is a life-threatening condition with dramatic burdens in terms of symptomatology, morbidity, and mortality [4]. The length of hospitalization of these patients is on average 5 days, and the readmission rate in the first 3 months after discharge can reach 30% [5,6]. In addition, in-hospital mortality is estimated at 10% of cases, and up to 20% of survivors die within 30 days [3].

AHF is a complex syndrome which requires emergency evaluation and treatment, being defined either as a rapid “de novo” onset or worsening of symptoms and signs of a previously stable chronic HF (CHF). Most patients are admitted for an acute decompensation of a CHF caused by precipitating factors, such as myocardial ischemia, uncontrolled arterial hypertension, arrhythmias, pulmonary embolism, acute kidney injury, infections, or low therapeutic adherence, while the “de novo” onset of AHF is mainly associated with acute coronary syndromes [6,7,8].

The relatively modest improvement concerning the therapeutic management of patients with AHF could be mainly due to the inability to recognize the various pathophysiological processes that occur during decompensation [9]. The diagnostic challenges arise from the fact that these patients exhibit a complex yet not specific clinical picture and usually have multiple comorbidities requiring a holistic approach [4,6,7,10].

Biomarkers can play an important role in addressing these deficiencies, and their routine incorporation into clinical practice can improve the outcomes. Many biomarkers are well established for their use in cardiovascular disease and in the management of CHF. In addition, several new biomarkers have recently shown promising results, especially in AHF, which could further improve patient management [9,11].

Current paradigm is shifting towards the study of various biomarkers which are associated with myocardial injury processes, neurohormonal activation, oxidative stress, and cardiac remodeling, with promising results in terms of clinical utility [5,11,12].

Endothelin-1 (ET-1) is a vasoconstrictive peptide that is primarily produced by endothelial cells and cardiomyocytes. It plays a role in the pathophysiology of various diseases, including heart failure, by causing cardiac hypertrophy and having profibrotic and proinflammatory effects [13,14,15,16]. Numerous literature data confirm that patients with these pathologies have higher levels of ET-1, which is linked to higher mortality as well as hospitalization rates. Based on previous observations, ET-1 may provide additional prognostic information beyond clinical variables [17].

The aim of this narrative review is to explore the pathophysiology and utility of ET-1 for the clinical diagnosis, prognosis and management in AHF, in order to determine whether ET-1 can be considered as a biomarker for these patients.

## 2. The Endothelin System: Morphofunctional Considerations

The endothelium has an essential role in protecting the arterial wall by releasing nitric oxide (NO) and prostacyclin. One year after discovering that endothelium has the ability to constrict and relax, one proposed the hypothesis of an endothelium-derived constricting factor [17]. The discovery of ET-1 by Yahagisawa et al. in 1988 represented a fundamental landmark in the field of cardiovascular research. In 1990, two receptors of ET-1 were identified, type A (ETA) and type B (ETB), respectively, offering the foundation for designing Bosentan (an antagonist of the ET-1 receptor), which can be currently used in the treatment of patients with pulmonary arterial hypertension. Multiple molecular and pharmacological approaches have outlined ET-1 as the most potent vasoconstrictor identified in biological systems to date. Since its discovery, remarkable efforts have been made to thoroughly understand the pathophysiological implications of this peptide in the cardiovascular system [18,19,20].

The ET system consists of three interconnected peptides: ET-1, ET-2 and ET-3. The 21-amino-acid peptide ET-1 is the main isoform produced at cardiovascular level and it has been the most intensively studied [19].

ETs have multiple pathophysiological functions involved not only in cardiovascular disorders (HF, arterial hypertension, cardiac hypertrophy, atherosclerosis), but also in pulmonary and kidney pathologies. Moreover, the ET system is implicated in tumoral processes, wound healing, and neurohormonal activation [21].

**Figure 1:**
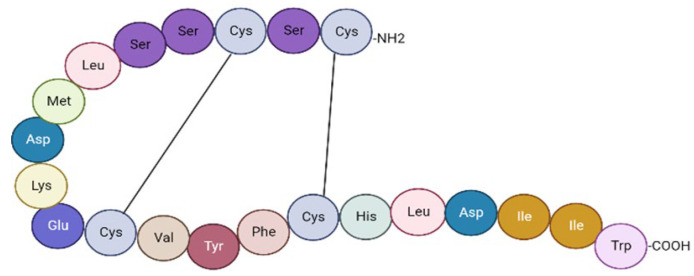
Endothelin-1 structure (adapted from Khimji et al. [21]) This image was created with BioRender (https://biorender.com/ accessed on 14 May 2023).

ET-1 is a 21-amino-acid cyclic peptide with two disulfide bonds consisting of four cysteines. The N-terminal end of the peptide defines its binding affinity to the receptor, whereas at the C-terminal end is located in the amino acids that bind to the receptor [21] (Figure 1).

**Figure 2:**
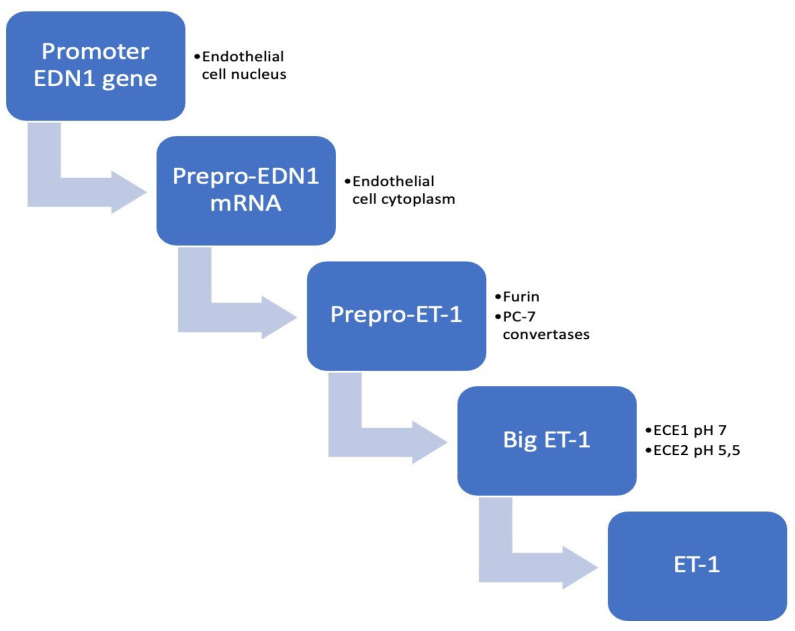
Endothelin-1 cycle. Abbreviations: EDN1—encoding endothelin-1; ECE—endothelin-converting enzyme; ET-1—endothelin-1.

In healthy tissues, ET-1 is produced in small amounts, keeping a balanced ratio between vasoconstriction and vasodilatation. However, under certain pathological conditions, the production of ET-1 is overstimulated by various regulating factors such as hormones, interleukins, cytokines, prostacyclin, NO, thrombin and mechanical stimuli. The production of mature ET-1 consists of two proteolytic steps. First, the coding gene for human ET-1 is converted under the action of regulating factors in a large precursor peptide known as pre-proET-1. This will be further cleaved in big ET-1 by specific furin-like proteases. Afterwards, the big ET-1 molecule is once more cleaved into the biologically active form by ET-converting enzymes (Figure 2).

It is interesting to note that ET-1 seems to be the only representative of the endothelin family produced by the endothelium. ET-1 acts as an autocrine/paracrine hormone and its plasma half-life is under 2 min. As a result, the interpretation of the ET value requires special attention. ET-1 is synthetized in a plethora of cell types, such as endothelial cells, cardiac myocytes, vascular smooth muscle cells, renal epithelial cells, inflammatory cells, neurons, and hepatocytes. ET-2 is secreted by both gastrointestinal and kidney cells, while ET-3 is produced in neural tissue (Table 1) [18,19,21,22].

ET-1 is an endogenous neurohormone that exerts its action by binding to specific receptors. At the vascular level, it causes vasoconstriction and cell proliferation via ETA receptors from vascular smooth muscle cells and cardiac myocytes, while activation of ETB receptors located on endothelial cells plays a role in vasodilatation. In addition, stimulation of ETB receptors located in vascular smooth muscle cells causes vasoconstriction [18,19,23].

## 3. ET-1 Implications in the Pathophysiological Mechanisms of AHF

**Figure 3:**
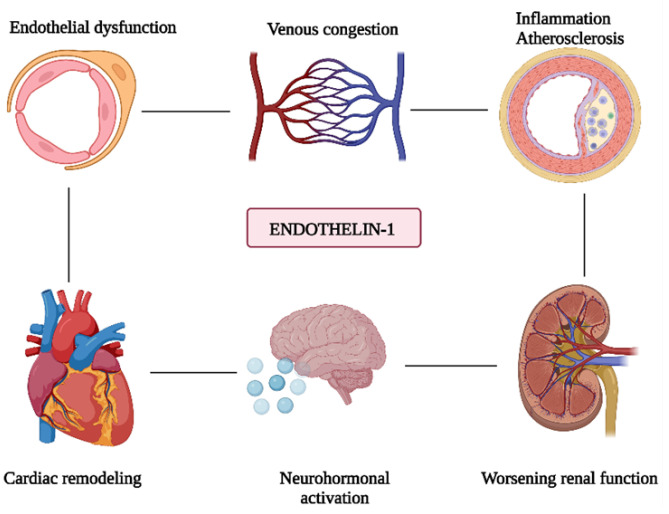
Pathophysiological mechanisms of AHF involving ET-1. This image was created with BioRender (https://biorender.com/ accessed on 14 May 2023).

ET-1 appears to be involved in multiple aspects of AHF pathogenesis (Figure 3), including decreased cardiac output, vasoconstriction, and neurohormonal activation. It induces marked systemic, pulmonary, coronary, and renal vasoconstriction, thus contributing to an increase in systemic and pulmonary vascular resistances, and in left ventricular filling pressures. In addition, it promotes sodium and fluid retention, and contributes to the occurrence of renal dysfunction [3,24]. ET-1 also participates in myocardial ischemia, and plays a central role in ventricular remodeling by directly stimulating myocardial hypertrophy and promoting fibrosis at this level [21].

### 3.1. Endothelial Dysfunction

The endothelium is a cellular monolayer that lines the whole circulatory system and plays an important role in its regulation by producing a considerable number of biologically active substances that participate in vascular tone regulation, cell growth, inflammation, and thrombosis/hemostasis. The healthy endothelium is a dynamic organ that maintains vascular tone by balancing the production of vasodilator and vasoconstrictor factors in response to a range of stimuli [22]. However, endothelial dysfunction refers to a pathological state where there is an imbalance in the major endothelial mechanisms [22,25].

The endothelium produces NO, the primary mediator of optimal vascular activity. Endothelial dysfunction causes increased synthesis and biological activity of ET-1 that oppose the vasodilator effects of NO. ET-1 acts as a strong vasoconstrictor and plays a crucial role in maintaining the vascular tone stability in both the overall circulatory system and the coronary arteries. Due to their opposing yet complementary roles, it is not surprising that NO and ET-1 are co-regulated by the same factors. Biomechanical stimuli like increased hydrostatic pressure and circumferential wall stress promote an intracellular signaling cascade that activates endothelial cells and their pro-oxidant, pro-inflammatory and vasoconstrictor properties [22,26,27,28,29].

In patients with HF, endothelial dysfunction is primarily attributed to elevated production of superoxide radicals and other oxidant species within the vasculature. This state of “oxidative stress” disrupts the equilibrium between the generation of oxygen free radicals and their neutralization by endogenous antioxidant mechanisms. Consequently, there is direct inactivation of NO, leading to impaired endothelial function. Apart from its involvement as a pathophysiological mechanism HF, endothelial dysfunction also serves as a prognostic indicator. This process is associated with a higher risk of hospitalization, cardiac transplantation, or death in patients with HF [30].

### 3.2. Venous Congestion

Venous congestion (VC) plays a major role in the development of AHF. However, the biomechanical consequences of VC on vascular endothelium neurohormonal activation and renal and cardiac failure, in particular, are still largely unstudied [31,32].

Multiple studies have clearly highlighted the causal relationship between VC and plasma ET-1 concentration. VC and increased plasma ET-1 levels are common in severe AHF. In addition, their levels decrease as the clinical condition improves [33]. In a study that included 24 healthy subjects, the researchers proved that the experimentally induced severe VC causes an immediate rise in plasma ET-1 levels and ET-1 expression in venous endothelial cells [34]. On the other hand, this model did not accurately simulate the hemodynamic conditions of peripheral congestion in HF patients. Therefore, the authors tried to understand whether lower VC levels might result in higher ET-1 levels, and if so, how rapidly these changes occur and whether or not they are sustained in the context of decongestion. In a more recent study, Lin et al. created a new model using acute experimental VC at comparable levels to those seen clinically in congestive conditions and observed an increase in ET-1 concentrations that were dependent on the VC dose and duration. In addition, ET-1 levels declined after 60 min of implementing the decongestion therapy, but remained significantly increased compared to the baseline [33].

### 3.3. Atherosclerosis and Inflammation

Acute myocardial infarction is the leading cause of HF [35,36]. During an episode of acute myocardial infarction, necrotic cardiac myocytes release ET-1 into the bloodstream, leading to an elevation in ET-1 levels. Increased concentrations of ET-1 were observed in both patients with ST-elevation acute myocardial infarction (STEMI) and non-ST-elevation acute myocardial infarction (NSTEMI). Moreover, ET-1 can predict in-hospital adverse cardiac events for these patients. Circulating ET-1 increases early in the course of angina and remains elevated until 72 to 96 h later. An elevated level of ET-1 has been linked to the expansion of myocardial necrosis, increased infarct size, and the development of ventricular pump failure subsequent to STEMI. Furthermore, this biomarker is associated with coronary microvascular dysfunction, which occurs during the reperfusion with percutaneous coronary intervention. Even after successful reperfusion strategy, the levels of this biomarker remain elevated [29,37,38].

Ischemic HF is the result of cardiomyocyte death and the formation of scar tissue. This process then leads to neurohumoral activation and ventricular remodeling [39]. Considering all of the above observations, it seems reasonable to assume that ET-1 concentrations are increased in ischemic AHF patients. The underlying pathophysiological mechanism of acute coronary syndromes is atherosclerosis. ET-1 appears to be implicated in the pathogenesis of atherosclerosis from an early stage. Additionally, the release of ET-1 is increased at different phases of atherosclerotic plaque evolution. Moreover, it was demonstrated that there is a substantial relationship between serum ET-1 values and the number of atherosclerotic lesions in these patients [19]. In agreement with these data, in vitro and animal studies have confirmed the involvement of ET-1 in the atherogenesis process. Dyslipidemia is one of the causal factors of atherosclerosis. An important finding is that hypercholesterolemia is correlated with higher ET-1 values and impaired endothelium-dependent vasodilation. Despite significant clinical and experimental evidence that ET-1 is involved in the atherosclerotic process, it remains unclear whether ET-1 represents a key pathogenic component in atherogenesis or just a marker of damaged vascular endothelium [40,41].

Inflammation is a crucial factor in the pathogenesis of AHF, contributing to disease progression and adverse outcomes. Apart from its implication in endothelial dysfunction, ET-1 is involved in the inflammatory mechanisms that take place in the arterial wall. ET-1, in particular, has been shown to activate macrophages even at small concentrations, resulting in the production of pro-inflammatory and chemotactic mediators such as tumor necrosis factor (TNF), interleukin (IL)-1 and IL-6, which are important in the atherogenesis process [42,43,44,45]. These cytokine cascades are believed to contribute to the development of clinical HF by causing endothelial dysfunction, pulmonary edema, and left ventricular dysfunction. ET-1 increases the expression of adhesion molecules on TNF-stimulated vascular endothelial cells and promotes polymorphonuclear neutrophil aggregation. ET receptor inhibition, on the other hand, reduces neutrophil accumulation and myeloperoxidase activity in the ischemic myocardium [22,46].

Further research confirmed the strong connection between ET-1, inflammation and atherosclerosis. Therefore, ET-1 is implicated in endothelial cell damage, release of pro-inflammatory factors, accumulation of lipids, and production of proteoglycans and collagen. ET-1 from the vascular smooth muscle stimulates the release of IL-6, causing an increase in oxidative stress with the production of reactive oxygen species, further stimulating the progression of atherosclerosis [22,45,47]. A potential predictor and mediator of atherosclerotic cardiovascular disease is the C-reactive protein (CRP). According to Verma et al., CRP enhances the expression of adhesion molecules and monocyte chemoattractant protein-1 in endothelial cells. Notably, bosentan and an anti-IL-6 antibody suppressed this effect, indicating that ET-1 and IL-6 are involved in the pro-inflammatory action of CRP [48].

### 3.4. Cardiac Remodeling

Cardiac remodeling plays a crucial role in the progression of HF and involves changes in the structure and function of the heart chambers. These changes include impaired systolic and diastolic function, reduced myocardial contractility, cardiac enlargement and hypertrophy. The mechanisms that initiate and maintain this remodeling process are represented by fibrosis, apoptosis, inflammation, oxidative stress and neurohormonal activation [49,50].

In reaction to pathophysiological stressors, cardiac hypertrophy is viewed as a compensatory mechanism that temporally preserves pump function. Untreated hypertrophy; however, can cause HF and sudden death [51]. Several studies have demonstrated that pressure overload and some endogenous compounds can induce myocardial hypertrophy and subsequent fibrosis. Among these substances, ET-1 has been studied with great interest because of its connection to stretch-induced inotropic, hypertrophic and pro-fibrotic responses [52,53].

Subsequently, several studies have indicated that cardiac fibrosis can be prevented or reduced with bosentan—a mixed ETA/ETB receptor antagonist—or ETB blockade, suggesting that the ET system can participate in the development of this pathological mechanism [54,55].

ET-1 is considered an important autocrine and paracrine regulator of cardiac function, with implications in the development of cardiovascular disease. ET-1 is produced by cardiomyocytes as a result of the action of neurohormonal and mechanical factors, and it rapidly modifies myocardial contractility. Long term, ET-1 expression and activity is increased, thus both inducing and sustaining pathological cellular responses in HF, ischemia and left ventricular hypertrophy [56]. As mentioned, ET-1 can affect muscle contractility and cause arrhythmias within minutes. On the other hand, this peptide can have a long-term effect in stimulating the growth of cardiomyocytes and cause hypertrophic cardiac remodeling, which later lead to HF. Therefore, ET-1 is thought to have a role in the etiopathology of these disorders. Furthermore, the plasmatic level of this biomarker is strongly connected with the severity of the cardiovascular disease, thereby making it an effective prognostic marker of impending HF [56,57]. Selvais et al. assessed 109 patients with HF and found a significant increase in plasma concentrations of ET-1 in functional class III to IV compared to patients with functional class II [58].

Numerous studies have demonstrated the predominance of ETA receptors on cultured neonatal and adult cardiomyocytes, while a combination of ETA and ETB receptors were observed on cardiac fibroblasts [59,60,61]. Experimental research showed that ET-1 synthesis is increased in animal models with cardiac hypertrophy and HF. An interesting finding is that in these animal models, chronic administration of ET receptor antagonists (ETA selective or mixed antagonists) reduces the development of myocardial hypertrophy [62]. Furthermore, there have also been studies highlighting the role of ET-1 in human cardiac remodeling, where it was proven that patients with hypertrophic cardiomyopathy had raised concentrations of this biomarker compared to control subjects [63].

Recently, there has been a growing focus on the topic of atrial cardiomyopathy. The concept of atrial cardiomyopathy includes functional, electrical and anatomical remodeling, the latter being characterized by left atrial dilatation and fibrosis [64]. It has already been proven that ET-1 promotes heart hypertrophy and fibrosis by activating fibroblast proliferation, the superoxide-metalloproteinase 9 cascade, and extracellular matrix synthesis [15,65]. Interestingly, it has been reported that atrial fibroblasts have greater sensitivity compared to ventricular fibroblasts when exposed to various pro-fibrotic stimuli [66]. According to Mayyas et al., ET-1 and its receptors were detected using immunostaining in both fibroblasts and atrial myocytes. The authors highlighted that ET-1 is associated with atrial dilatation, fibrosis and hypertrophy. Notably, increased atrial ET-1 level is linked to enlarged left atrial size and the presence of HF. Moreover, ET-1 has been demonstrated to influence intracellular calcium (Ca^2+^) release, resulting in elevated intracellular Ca^2+^ levels and premature atrial beats. This has significant implications for the electrical remodeling of the atrial myocardium [67].

Considering all of the abovementioned details, we can assume that ET-1 has a significant role in the process of cardiac remodeling in HF patients.

### 3.5. Worsening Renal Function

The connection between the heart and kidneys can be demonstrated by defining the cardiorenal syndrome (CRS). The term CRS is frequently used to describe the detrimental consequences of diminished renal function on the cardiovascular system, despite being widely recognized as a disorder characterized by the development of renal insufficiency subsequent to HF. In summary, the CRS is a complex bidirectional syndrome, where the principal failing organ is either the heart or the kidney [68,69,70,71].

The pathophysiological mechanisms that are implicated in the development of renal injury in the presence of AHF are increased venous pressure, inflammation, endothelial activation and neurohormonal activation. Frequently, patients with AHF already have chronic renal dysfunction, which makes them more prone to acute kidney injury [72]. The aggravation of the renal disfunction in AHF is another factor associated with a poorer prognosis [73,74,75,76,77].

Many studies have shown the importance of the ET system in renal physiology and pathophysiology. ET-1 seems to be involved in the development of chronic kidney disease (CKD), acute renal injury and renal remodeling. To start with, the production of ET-1 is increased in CKD, and its plasma levels correlate with the severity of renal dysfunction. Secondly, multiple aspects of renal function are regulated by ET. ET-1 is involved in regulating renal blood flow and glomerular filtration rate, sodium and water excretion, acid-base balance. Notably, the administration of ET-1 in humans results in an important decrease in renal blood flow, glomerular filtration as well as diuresis. It is known that ET-1 can induce fibrosis, inflammation and cell proliferation. Moreover, this neurohormone can exert these effects on the majority of the kidney’s cell types. Thirdly, in several CKD models, ET receptor antagonists delay the onset and slow the course of the illness [21,78,79]. All things considered, we can conclude that the ET system has an important role in renal pathology.

### 3.6. Neurohormonal Activation

Multiple studies have demonstrated that neurohormonal activation plays a central role in cardiac performance and HF pathology. As a response to the decreased cardiac output in HF, compensatory mechanisms like neurohormonal activation are involved to ensure optimal blood pressure and perfusion to different vital organs such as the brain, kidneys and lungs. Even if these adjustments are helpful in the early stages of the disease, when HF progresses, they can have a detrimental impact on the cardiac workload, ultimately causing acute decompensated HF (ADHF) [1,80].

Due to decreased cardiac output and renal blood flow, HF is characterized by the activation of the following neurohormonal factors: ET-1, renin-angiotensin-aldosterone system, sympathetic nervous system and anti-diuretic hormone. Consequently, the levels of ET-1, angiotensin II and norepinephrine are elevated in patients with AHF [34,81]. Activation of the renin–angiotensin–aldosterone system in the early stages of HF activates adaptive responses with the purpose of preserving perfusion. These changes involve increased myocardial contractility, salt and fluid retention, and peripheral vasoconstriction. However, as time progresses, these adaptive mechanisms become detrimental, leading to worsening cardiac and end-organ dysfunction [1]. Regarding the role of sympathetic nervous system in the pathophysiology of HF, sustained catecholaminergic stimulation causes myocardial cardiotoxicity and disease progression, with systolic dysfunction and arrhythmogenic risk [82].

Moreover, accumulating evidence suggests that neurohormonal activation can have prognostic value when it comes to mortality from HF. A substudy of the Prospective Randomized Evaluation of Cardiac Ectopy with Dobutamine or Nesiritide Therapy (PRECEDENT) study analyzed the complete neurohormonal and cytokine profile at the time of admission for 88 patients with HF. In this research, the authors investigated whether some neurohormonal biomarkers (ET-1, aldosterone, norepinephrine, plasma renin activity) and cytokines (TNF-α, IL-6) could predict survival in patients hospitalized for ADHF. This study demonstrated that ET-1 measurement offers significant predictive information on the risk of mortality following hospital discharge. An interesting fact is that ET-1 appears to have a higher discriminatory power than other neurohormones and cytokines for risk stratification in HF [83].

## 4. Endothelin-1: A Promising Biomarker in AHF

At this time, we have a better understanding of the role played by ET-1 in the pathophysiology of HF, and it seems reasonable to assume that this neurohormone could be a promising biomarker in AHF. Considerable efforts have been made in the last three decades to demonstrate the potential diagnostic, prognostic and therapeutic target roles of ET-1 in HF.

Various animal studies have shown that ET-1 concentrations are elevated in HF models. A study demonstrated that in rats with HF, the production of ET-1 and the number of myocardial ET receptors are significantly increased [84].

Since its discovery, numerous investigations on the contribution of ET-1 in AHF patients beyond the traditional biomarkers have been conducted. The severity of symptoms and cardiac dysfunction in AHF is correlated with the plasma level of ET-1, which can be used as a prognostic indicator for these patients. In clinical trials, ET-1 has been shown to be predictive of life-threatening arrhythmias, hospitalization, and patient mortality [21,23,85].

A recent study confirmed that ET-1 is elevated in AHF and decreases with the therapeutic stabilization of patients within 30 days. Secondly, plasma ET-1 was independently correlated with 180-day mortality, providing additional prognostic information over that obtained by N-terminal pro-B-type natriuretic peptide (NT-proBNP). Thirdly, persistence of elevated levels of this biomarker 48–72 h after admission was associated with increased in-hospital mortality or aggravation of HF. Therefore, the increase in ET-1 values may have an important role in identifying patients with AHF and pose an increased risk for unfavorable short-term as well as long-term evolution [86].

In a study that used a multibiomarker profile including ET-1, NT-proBNP, high-sensitivity cardiac troponin (hs-cTn) I, soluble suppression of tumorigenicity-2 (sST2) and galectin 3 for the clinical assessment of 115 patients with HF, ET-1 was correlated with a more advanced HF, higher pulmonary pressure and reduced right ventricular function. The authors confirmed that when combined with other biomarkers in a multimarker profile, ET-1 may be a special predictor of the prognosis of HF. In addition, serial ET-1 measurement might offer further prognostic information [87]. Another clinical study indicated that ET-1 level had a substantial and independent contribution in predicting the long-term cardiac mortality in patients with HF [58]. These findings are supported by another large research that included 1653 patients with AHF enrolled in the PROTECT trial. Forty-three novel and already established biomarkers were serially evaluated to determine their performance in predicting early post-discharge death or rehospitalization. Among these, ET-1 identified the highest number of high-risk patients. Although further investigation is required, ET-1 may be the ideal individual biomarker for high-risk prediction of early post-discharge events in AHF [88,89,90]. Furthermore, Zymliński et al. confirmed the association between elevated ET-1 levels and a higher risk of 1 year mortality in AHF patients [91].

Besides ET-1, similar peptides formed during its production from the precursor molecule pre-proendothelin-1 have been extensively researched as possible risk indicators for cardiovascular events. Multiple studies have shown that the plasmatic concentration of big ET-1 is correlated with the outcome in patients with HF. Pacher et al. investigated the hypothesis that high levels of the precursor of ET-1 may be related to poor outcomes in advanced HF. This statement seems reasonable since the elevation of ET-1 is mainly connected to its precursor, big ET-1. The authors have demonstrated that the plasmatic concentrations of this peptide were higher in the severe forms of HF and had a significantly prognostic importance [92]. Notably, a previous study clearly established that an increase in plasma ET in severe human CHF is mostly due to an increase in big ET-1. Additionally, the authors indicated that an increased ET concentration is representative for severe HF [93]. In accordance with other research papers, a study involving 2359 participants enrolled in the Valsartan Heart Failure Trial provided further proof that the big ET-1 level is associated with the severity of the disease and that it can be considered an independent prognostic marker [94]. Once again, the strong predictive performance of ET-1 and big ET-1 in advanced HF was confirmed by Beneden et al. [95]. Furthermore, recent results from a study conducted by Mo et al. revealed that big ET-1 was correlated with the risk for short-term adverse events in ADHF patients [24]. Basically, all of this evidence points towards the promising role of ET1 as a clinically useful biomarker in AHF.

Nevertheless, there are certain categories, such as patients with autoimmune diseases, who require special consideration in diagnosing AHF based on ET-1 levels, as elevated concentrations of ET-1 is a common finding in both conditions. Moreover, the overlapping increase in ET-1 levels poses a challenge in distinguishing between AHF and the cardiac involvement seen in autoimmune disorders such as rheumatoid arthritis and systemic lupus erythematosus. When evaluating autoimmune disease patients with potential HF-related symptoms, it is essential to carefully consider the clinical context, thereby conducting a comprehensive physical examination, and making use of the full spectrum of diagnostic tools (i.e., cardiac biomarkers, autoantibody panels, inflammatory markers, and echocardiography). These measures are essential for accurately identifying AHF and successfully managing these complex cases [96,97,98,99]. Moreover, ET-1 holds potential as a valuable biomarker in guiding therapeutic strategies. Previous studies have indicated that ET-1 plays a role in neurohormonal activation mediated by angiotensin II. It is intriguing to consider whether patients with elevated ET-1 levels may experience enhanced benefits from angiotensin-converting enzyme inhibitor (ACEI) administration. Gaggin et al. observed an inverse correlation between ET-1 levels and changes in ACEI dose. Patients with ET-1 values above the median tended to have lower baseline doses of lisinopril [87]. In patients with HF with preserved ejection fraction (HFpEF) and HF with mid-range ejection fraction (HFmrEF), increasing endothelial function is highly connected to improved outcomes and functional class. Perindopril is the only ACEI that has been shown to improve endothelial function. Notably, Safonova et al. demonstrated that after 12-month therapy with perindopril, ET-1 level decreased significantly in both HFpEF and HFmrEF patients [100]. In addition, other studies have shown that captopril and lisinopril caused a significant reduction in ET-1 production [101,102]. Other therapies have been proposed to reduce ET-1 concentrations. Beta-blockers such as carvedilol, nebivolol, metoprolol and propranolol can also decrease the production and release of ET-1 in human endothelial cells [103,104]. Furthermore, loop diuretics like furosemide and torasemide may help improve endothelial function and potentially lead to a decrease in ET-1 levels [105]. However, no significant correlations were found between changes in ET-1 levels and the use of angiotensin receptor blockers or mineralocorticoid receptor antagonists [87,106].

Clinical evidence supports the beneficial effects of nesiritide in the treatment of ADHF. Its use has been shown to reduce systemic vascular resistance, right atrial pressure, pulmonary capillary wedge pressure, and mean pulmonary arterial pressure. Aronson et al. showed that administration of both low and high doses of nesiritide therapy led to a significant reduction in plasma ET-1 levels. Nesiritide’s capacity to suppress ET-1 may be another mechanism through which it has favorable hemodynamic and clinical benefits in individuals with HF [107].

Another interesting finding relates to treatment with sodium-glucose cotransporter-2 (SGLT-2) inhibitors in HF. A current study has demonstrated that the beneficial effects of dapagliflozin remained consistent regardless of the initial ET-1 levels, and treatment with dapagliflozin resulted in a modest reduction in serum ET-1 concentration. The observed reduction in ET-1 levels with dapagliflozin treatment provides insights into a potential novel mechanism of action of SGLT-2 inhibition. Further investigation is needed to fully elucidate the precise mechanisms underlying this interaction and its clinical implications in the management of HF [17]. Table 2 summarizes the effects of various drugs utilized in the treatment of AHF on ET-1 levels.

In patients with advanced HF, cardiac resynchronization therapy (CRT) has been shown to enhance survival and quality of life. However, a significant portion of patients receiving CRT devices do not experience the anticipated clinical benefits. Therefore, there is a need for reliable predictors to identify high-risk patients who would benefit from priority treatment. In a recent study that included 367 patients who received CRT, the authors evaluated the impact of serum big ET-1 levels on all-cause mortality, as well as the rates of heart transplantation and cardiac hospitalization. A baseline level of big ET-1 greater than 0.56 pmol/L was found to be independently associated with increased all-cause mortality and HF hospitalization among patients receiving CRT. These findings suggest that incorporating big ET-1 into the marker panel for risk stratification can help identify high-risk CRT patients who would benefit from priority treatment [108]. In support of this evidence, another study found that big ET-1 can be a predictor of all-cause mortality and heart transplantation risk in patients with non-ischemic cardiomyopathy and CRT [109].

An essential aspect in the management of HF patients is the prevention of life-threatening tachyarrhythmias and sudden cardiac death. The placement of an implantable cardioverter–defibrillator (ICD) is a common procedure for primary prevention. Levels of plasma big ET-1 have shown predictive value for ventricular arrhythmias and adverse outcomes in patients who are candidates for primary prevention ICD therapy. Therefore, measuring big ET-1 could potentially assist in risk stratification for ICD implantation in these patients [110].

## 5. Multimarker Panels Incorporating ET-1 and Conventional Biomarkers in AHF

By analyzing multiple biomarkers simultaneously, multimarker panels provide a more holistic and detailed picture of the disease process. This enables healthcare professionals to better risk-stratify patients, tailor treatment strategies, and predict prognosis in AHF. The incorporation of ET-1 into multimarker panels holds particular promise, as it has shown associations with disease severity, cardiac remodeling, and clinical outcomes in AHF. Utilizing multimarker panels in AHF has the potential to enhance diagnostic accuracy, optimize therapeutic interventions, and improve patient outcomes in this challenging clinical setting. Current European (ESC) and American (American College of Cardiology/American Heart Association, ACC/AHA) guidelines include established biomarkers like NT-proBNP, B-type natriuretic peptide (BNP), and hs-cTn in AHF patient assessment [111].

### 5.1. Natriuretic Peptides and ET-1

Among AHF biomarkers, natriuretic peptides have been the most extensively studied and validated molecules, and are considered a benchmark for all other markers. Their discovery and integration into clinical practice have demonstrated the substantial additive benefit that biomarkers have alongside other clinical information to diagnose, stratify risk, and manage patients with AHF [7,11,112].

Natriuretic peptides are influenced by certain demographic aspects and numerous pathological conditions such as pulmonary thromboembolism, rhythm disorders, valvulopathies, anemic syndrome and renal dysfunction, which cause elevated serum concentrations [10]. Regarding the factors that influence ET-1, it is widely recognized that its concentrations are elevated in the presence of renal dysfunction [113].

The degree of cardiac dysfunction and the severity of symptoms are correlated with serum concentrations of natriuretic peptides [5,11]. Likewise, ET-1 levels are associated with the functional capacity and severity of HF, as they are significantly higher in patients with moderate-to-severe form compared to those with mild symptoms [58]. In contrast to BNP, it is interesting to note that there was no relationship observed between ET-1 levels and left ventricular ejection fraction [113].

The evaluation of ventricular remodeling and cardiac function in clinical settings is primarily conducted using color Doppler ultrasound, which provides accurate insights into the condition of the heart. However, this method does not provide a comprehensive assessment of the overall health status of the organism. To expand the range of options available for assessing cardiac pump function and ventricular remodeling, a recent study examined the relationship between their related indexes and serum levels of BNP and ET-1 in HF patients. As expected, the findings revealed significant elevation of BNP and ET-1 levels in these patients compared to the control group. In addition, both biomarkers were negatively correlated with the cardiac pump related indexes and positively correlated with the ventricular remodeling related indexes. The observed results suggest that BNP and ET-1 levels can serve as reliable markers in HF, offering valuable insights for evaluating the medical condition of these patients. Furthermore, over the course of a 12-month follow-up, there was a notable increase in the levels of BNP and ET-1 in patients who encountered cardiovascular events, emphasizing the prognostic significance of these biomarkers [114].

The prognostic significance of ET-1 has shown mixed results in comparison to natriuretic peptides. The Val-HeFT study, which included 2359 patients, demonstrated that high levels of big ET-1 (precursor of ET-1) were associated with adverse outcomes and mortality. However, BNP exhibited greater prognostic value in this study [96]. In contrast, another interesting study, which included 109 fully treated patients with HF, revealed that from the evaluated parameters: ET-1 level, New York Heart Association (NYHA) class, N-terminal proatrial natriuretic factor level, BNP level, left ventricular ejection fraction and age, only ET-1 had a significant and independent impact on prognosis and was able to identify a specific subgroup of patients who had an exceptionally high risk of mortality [58]. In the ASCEND-HF biomarker substudy, ET-1 offered additional prognostic information, beyond what was provided by NT-proBNP, in hospitalized patients with AHF [86].

Independent of left ventricular ejection fraction, ET-1 has been found to be a significant predictor of adverse cardiovascular events and mortality [86]. Therefore, ET-1 has the potential to provide additional data when included in multimarker panels, allowing the assessment of disease advancement, particularly with regard to left ventricular remodeling and worsening symptoms.

### 5.2. Cardiac Troponin and ET-1

In patients with AHF, testing for cardiac troponin (cTn) I or cTnT is performed to confirm the existence of type 1 myocardial infarction or AHF-related injury. In the absence of acute myocardial ischemia, an increase in cTn should be interpreted as myocardial injury. It has been proven that levels of hs-cTn are commonly elevated in patients with HF, regardless of the presence or absence of coronary artery obstruction [101]. This statement is supported by Pascual-Figal et al. in a study that included 202 patients admitted with ADHF and without criteria for acute myocardial infarction. They demonstrated that 98% of the participants had detectable levels of hs-cTnT, of which 81% were higher than the upper normal limit [115].

Since hs-cTn concentrations indicate a higher risk of mortality and a higher probability of progressive ventricular remodeling [111], including ET-1 and hs-troponin in a multimarker panel could provide comprehensive information about the underlying pathophysiological processes and prognosis.

The potential cause of cTnI elevations in AHF is myocyte damage. Other theories that include, apoptosis, or necrosis have also been proposed to explain the elevation in hs-cTn in HF patients [116]. There are also studies that highlighted the prognostic value of cTn in patients with AHF. For instance, in a study by Pascual-Figal et al. [117] concentrations of hs-cTnT remained a strong and independent predictor of all-cause mortality in one study, even after adjusting for multivariable factors such as natriuretic peptides and sST2. In addition, cTn concentrations may also be helpful in predicting outcomes during hospitalization and after the release from the hospital and administration of treatment for AHF [118,119,120]. The largest study, ADHERE, which examined the cTn value in patients with AHF, both ischemic and non-ischemic, provided incontrovertible evidence of an increased in-hospital mortality rate in patients with a high cTn level. The simultaneous measurement of ET-1 and hs-cTn levels could allow for a more comprehensive evaluation of cardiac function, myocardial damage, and vascular abnormalities, providing clinicians with important insights for individualized treatment strategies and monitoring of disease progression [121].

## 6. Multimarker Panel Incorporating ET-1 and Novel Biomarkers in AHF

### 6.1. Growth Differentiation Factor-15 and ET-1

Growth Differentiation Factor-15 (GDF-15) is an emerging biomarker that is involved in various pathophysiological pathways in HF, including fibrosis, remodeling, and oxidative stress. Furthermore, GDF-15 is involved in the development of endothelial dysfunction by disrupting the normal functioning of NO-dependent vascular systems and promoting excessive proliferation of endothelial cells, those pathways being commonly shared with the ET-1. Moreover, GDF-15 may influence ET-1 by accelerating endothelial senescence via the activation of various pro-oxidant pathways. As a result, these processes lead to significant synthesis of reactive oxygen species, thus inducing changes in the structure and function of the vascular endothelium, and further contributing to cardiac injury [12].

The findings regarding the diagnostic and prognostic value of GDF-15 in AHF are remarkable. In a recent study, Miftode et al. reported that GDF-15 consistently showed a diagnostic performance comparable to that of NT-proBNP, which is considered the current gold standard. In addition, multiple studies have highlighted the prognostic significance of elevated GDF-15 serum levels, as they have shown strong correlations with increased mortality. In fact, some authors suggest that GDF-15 may be a more powerful predictor of outcomes compared to classical biomarkers (NT-proBNP, hs-cTn) [12,122,123,124].

To the best of our knowledge, there are currently no studies in the literature that have used a multimarker approach incorporating both ET-1 and GDF-15 for the assessment of patients with AHF. Given the shared involvement of ET-1 and GDF-15 in fibrosis, cardiac remodeling and endothelial dysfunction, further exploration of the association between these two biomarkers presents an intriguing and worthwhile avenue for extensive research.

### 6.2. Soluble Suppression of Tumorigenicity 2 and ET-1

In recent studies, sST2, a novel biomarker associated with cardiac remodeling and fibrosis, has shown promise as a predictive factor for long-term mortality in patients with ADHF. The prognostic value of sST2 is complementary to that of NT-proBNP, providing additional insights into the severity of ventricular remodeling and the hemodynamic status in these patients. Furthermore, elevated levels of sST2 may help identify HF patients at a higher risk of sudden cardiac death. These findings highlight the potential of sST2 as a valuable biomarker in risk stratification and prognosis assessment in ADHF patients [117].

While some studies suggest that there is no definitive correlation between elevated sST2 levels and impaired systolic function, they also highlight the role of sST2 in risk stratification of HF patients. Moreover, these studies emphasized the value of sST2 for diagnosing AHF in patients with dyspnea, regardless of whether they exhibit reduced or preserved ejection fraction [125,126,127,128]. Following a similar pattern, it has been demonstrated that the prognostic value of ET-1 is not influenced by left ventricular ejection fraction, indicating its independent significance in predicting outcomes in HF patients [113]. Notably, a common link between ET-1 and sST2 is that both biomarkers are associated with the NYHA functional class [58,129]. A frequent occurrence in individuals diagnosed with AHF is impaired renal function. In contrast with ET-1, sST2 is a more accurate diagnostic tool in patients with HF and concomitant kidney disease, because its value is not affected by the renal function [129].

An interesting multimarker panel was proposed by Gaggin et al. The authors observed that a multimarker profile incorporating traditional risk factors along with ET-1, NT-proBNP, hs-TnI, and sST2 demonstrates superior performance in predicting cardiovascular events, with ET-1 playing a significant role in improving risk stratification [87].

## 7. ET Receptor Antagonist Therapy in AHF

Soon after the discovery of the ET system, numerous studies were performed to explore whether blocking this pathway might become a beneficial treatment option for patients with HF. The inhibition of the ET receptors ETA and ETB through the use of ET receptor antagonists (ERA) is still being investigated. The VERITAS trial revealed that the treatment with non-selective tezosentan in AHF had minimal clinical consequences [3]. Despite the successful experimental studies, the results of clinical trials have been disappointing [19,130]. There are several causes that can explain these differences. Firstly, the administration of standard HF therapy simultaneously with ERA medication may cover the positive effect that ERA can have in patients with this disease—a situation that is certainly not present in the animal models that only received ERA. Secondly, another possible reason is that the ERA treatment has various effects and can induce undesired consequences in untargeted organs exclusively in humans. These findings suggest that there may be a gap in our understanding of the role of ET in HF pathophysiology. Further research is required to implement novel therapeutic strategies targeting the ET system or the underlying mechanisms of ET activation in HF patients [130]. On the other hand, ERAs have been demonstrated to be effective in treating pulmonary arterial hypertension (PAH) [131]. At the moment, the most often used ERAs are bosentan, ambrisentan, and macitentan, which specifically inhibit the actions of ET-1 on a variety of receptors. The first orally accessible medicine for the treatment of PAH was bosentan, a dual antagonist of ETA and ETB. When compared to placebo, it has been shown to enhance exercise capacity and different hemodynamic parameters in individuals with moderate and severe forms of PAH. Another medication that has been proven to improve exercise capacity and hemodynamic measures is alisentan, a highly selective ETA antagonist. In addition, macitentan belongs to a new class of dual ERAs with improved tissue penetration and receptor affinity. Studies have demonstrated that macitentan can considerably lower the mortality or hospitalization rate in patients with PAH and can enhance cardiac function, quality of life and NT-proBNP [132].

## 8. Future Perspectives

The diagnostic and prognostic role of ET-1 in AHF holds significant potential for future prospects. As our understanding of AHF continues to evolve, there is growing recognition of the complex mechanisms involved in its pathophysiology. ET-1 has shown potential as a biomarker for assessing disease severity, predicting outcomes, and guiding therapeutic strategies in this pathology. Further research is warranted to explore its utility in multimarker panels, where combining ET-1 with other conventional or novel biomarkers could enhance diagnostic accuracy and risk stratification. By incorporating ET-1 into comprehensive diagnostic algorithms, healthcare professionals may be able to identify high-risk patients, tailor treatment approaches, and improve clinical decision making. Additionally, ongoing studies aim to elucidate the dynamic changes in ET-1 levels during AHF exacerbations, which could provide insights into disease progression and response to treatment.

The use of ERA in HF patients has demonstrated efficacy in improving hemodynamics, exercise capacity, and clinical outcomes in certain patient populations. However, future perspectives on therapy with ERA extend beyond their current indications. It would be interesting to explore the potential benefits of ERA in specific subsets of HF patients, such as those with preserved ejection fraction, right HF or PAH. Additionally, the combination of ERAs with other targeted therapies, such as IECA, angiotensin receptor blockers or neprilysin inhibitors, should be investigated. Moreover, advancements in the development of selective ERA with improved safety profiles and reduced side effects may further expand their therapeutic potential. Overall, further clinical trials and research are warranted to establish their optimal role and benefits in different patient populations.

In summary, the use of ET-1 as a diagnostic and predictive tool in AHF offers promise for the future, with the potential to improve patient care, personalize medication, and improve overall outcomes.

## 9. Conclusions

AHF is the most common cause of hospitalization and is associated with a high risk of readmission and mortality. There is a growing interest in the field of novel cardiac biomarkers that increase the diagnostic and prognostic accuracy of these patients. ET-1 has a crucial role in the pathogenesis of HF. Multiple studies have demonstrated that the severity of symptoms and cardiac dysfunction in AHF is correlated with the circulating levels of ET-1, which can be used as a strong prognostic indicator for these patients. Furthermore, multimarker panels that include ET-1 might improve clinical assessment and risk stratification in patients with AHF. Unfortunately, regarding the treatment with ERA in the setting of AHF, the results of previous studies did not meet expectations. Although significant progress has been made since the discovery of ET-1, further research is necessary before implementing this biomarker in clinical practice.

## Figures and Tables

**Table 1:** Subtypes of ET and their tissue sites of synthesis.

ET-1	ET-2	ET-3
Vascular smooth muscle cells	Gastrointestinal stromal cells	Gastrointestinal stromal cells
Endothelial cells	Kidney epithelial cells	Kidney epithelial cells
Cardiac myocytes		Neurons
Kidney epithelial cells		Glia
Inflammatory cells		
Hepatocytes		
Neurons		

**Table 2:** Common medications in HF and their effect on the serum level of ET1.

Drug	ET-1 Level
**Loop diuretics** -Furosemide -Torasemide	**↓**
**Mineralocorticoid receptor antagonists** -Spironolactone	**↔**
**ACEI** -Perindopril -Captopril -Lisinopril	**↓**
**Angiotensin receptor blockers**	**↔**
**Beta-blockers** -Metoprolol -Propranolol -Nebivolol -Carvedilol	**↓**
**SGLT2 inhibitors** -Dapagliflozin	**↓**
**Vasodilators** -Nesiritide	**↓**

Legend: ACEI—angiotensin-converting enzyme inhibitors; SGLT2—sodium-glucose cotransporter-2; ↓—decreased level; ↔—no significant changes.

## Data Availability

Data sharing not applicable.
